# Exploring chemical space for “druglike” small molecules in the age of AI

**DOI:** 10.3389/fmolb.2025.1553667

**Published:** 2025-03-17

**Authors:** Aman Achuthan Kattuparambil, Dheeraj Kumar Chaurasia, Shashank Shekhar, Ashwin Srinivasan, Sukanta Mondal, Raviprasad Aduri, B. Jayaram

**Affiliations:** ^1^ Department of Biological Sciences, BITS Pilani K K Birla Goa Campus, Zuarinagar, Goa, India; ^2^ School of Interdisciplinary Research, Indian Institute of Technology Delhi, New Delhi, India; ^3^ Supercomputing Facility for Bioinformatics and Computational Biology, Indian Institute of Technology Delhi, New Delhi, India; ^4^ Department of Computer Science & Information Systems, BITS Pilani K K Birla Goa Campus, Zuarinagar, Goa, India; ^5^ Department of Chemistry, Indian Institute of Technology Delhi, New Delhi, India

**Keywords:** machine learning (ML), artificial intelligence, computer aided drug design (CADD), small molecules, BIMP

## Abstract

The announcement of 2024 Nobel Prize in Chemistry to Alphafold has reiterated the role of AI in biology and mainly in the domain of “drug discovery”. Till few years ago, structure-based drug design (SBDD) has been the preferred experimental design in many academic and pharmaceutical R and D divisions for developing novel therapeutics. However, with the advent of AI, the drug design field especially has seen a paradigm shift in its R&D across platforms. If “drug design” is a game, there are two main players, the small molecule drug and its target biomolecule, and the rules governing the game are mainly based on the interactions between these two players. In this brief review, we will be discussing our efforts in improving the state-of-the-art technology with respect to small molecules as well as in understanding the rules of the game. The review is broadly divided into five sections with the first section introducing the field and the challenges faced and the role of AI in this domain. In the second section, we describe some of the existing small molecule libraries developed in our labs and follow-up this section with a more recent knowledge-based resource available for public use. In section four, we describe some of the screening tools developed in our laboratories and are available for public use. Finally, section five delves into how domain knowledge is improving the utilization of AI in drug design. We provide three case studies from our work to illustrate this work. Finally, we conclude with our thoughts on the future scope of AI in drug design.

## 1 Introduction

Small molecule libraries play a pivotal role in modern drug discovery, serving as essential collections of chemical compounds for identifying molecules with desired biological activity (Dandapani et al., 2012; Saldívar-González et al., 2020). These libraries can be broadly categorized into diverse libraries, which offer broad structural variety, and focused libraries that target specific protein families or biological pathways, such as GPCR kinases (Dandapani et al., 2012; Harris et al., 2011). The generation of these libraries employs various methodologies, including combinatorial chemistry, diversity-oriented synthesis, fragment-based approaches, natural product extraction, and computational generation of virtual libraries (Dandapani et al., 2012; Saldívar-González et al., 2020; Sadybekov and Katritch, 2023).

The success of *in silico* drug design is significantly influenced by the selection of appropriate small molecule libraries through multiple factors (Dandapani et al., 2012). While diverse libraries enable broad exploration of chemical space, focused libraries can enhance hit rates for specific targets (Dandapani et al., 2012; Harris et al., 2011). The assessment of physicochemical properties and drug-likeness, particularly through established filters like Lipinski’s RO5 (Lipinski et al., 1997) ensures appropriate absorption, distribution, metabolism, and excretion characteristics (Dandapani et al., 2012; Sadybekov and Katritch, 2023). Aqueous solubility remains a critical factor, as poorly soluble molecules can lead to false positives and limited optimization potential.

**FIGURE 1:**
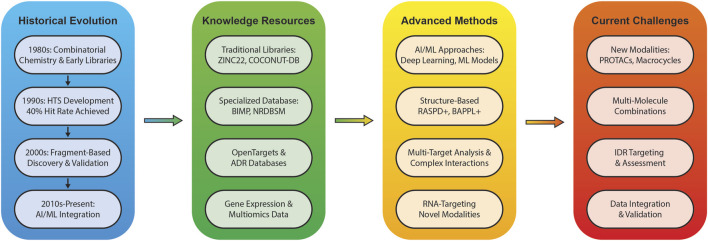
An overview of the evolution, current state, and future directions of small molecule drug discovery in the age of AI is presented in this Figure, highlighting the key developments from historical advances to emerging challenges.

These libraries find application across various drug discovery approaches. In virtual screening, libraries undergo computational assessment for target binding potential, often in conjunction with experimental screening to enrich compound collections (Dandapani et al., 2012; Sadybekov and Katritch, 2023). *De novo* drug design utilizes these libraries as foundations for generating novel molecules, particularly when existing libraries have been exhausted, incorporating target constraints and leveraging machine learning approaches (Sadybekov and Katritch, 2023; Chang et al., 2023). Fragment-based drug design employs libraries of small fragments to identify weak-binding molecules that can be elaborated into more potent compounds (Dandapani et al., 2012; Sadybekov and Katritch, 2023), while lead optimization uses libraries to enhance existing compounds’ properties through quantitative structure-activity relationship (QSAR) models (Sadybekov and Katritch, 2023; Chang et al., 2023). Figure 1 provides a comprehensive overview of how these approaches have evolved from historical developments to current AI-integrated methodologies, highlighting the interconnected nature of various tools and resources in modern drug discovery.

### 1.1 Historical context of small molecule libraries

The evolution of small molecule drug discovery has been marked by transformative technological advances since the 1980s. The field was revolutionized by combinatorial chemistry, progressing from Geysen’s multi-pin technology to the first small-molecule combinatorial library by Bunin and Ellman in 1992 (Liu et al., 2017). This advancement, integrated with high-throughput screening (HTS) and computational methods, became fundamental to pharmaceutical lead discovery by the late 1990s (Appell et al., 2001).

Screening methodologies evolved in parallel, with laboratory robotics enabling automated biological assays that could generate up to 100,000 data points daily through ultrahigh-throughput screening platforms (Appell et al., 2001). The field progressed from random to focused libraries, with discovery libraries decreasing from 57% (1992–1997) to 21% (1999) (Appell et al., 2001), in contrast to targeted and optimization libraries. Fragment-Based Drug Discovery (FBDD) emerged as a complementary approach, leading to FDA-approved drugs like Vemurafenib (2011) and Venetoclax (Mureddu and Vuister, 2022; Bon et al., 2022).

The success of this evolution is exemplified by landmark drugs such as Imatinib (Gleevec), which revolutionized chronic myeloid leukemia treatment in 2001 (Müller, 2009; Druker and Lydon, 2000). Venetoclax demonstrated the feasibility of targeting protein-protein interactions, representing one of the first non-natural product clinical agents in this space (Congreve et al., 2008; Murray and Rees, 2009). While recent trends show a shift towards biologics due to lower clinical trial attrition rates (Sun et al., 2011), small molecules continue to comprise approximately 40% of FDA approvals annually (Mureddu and Vuister, 2022). However, challenges persist, with only 1% of compounds progressing from discovery to approved New Drug Application (NDA), and a 50% failure rate in clinical trials due to ADME issues (Appell et al., 2001), emphasizing the ongoing need for innovative approaches in small molecule drug discovery.

### 1.2 Types of small molecule libraries

A fundamental distinction exists between physically synthesized and virtually synthesizable libraries. Synthesized libraries represent physical collections created through chemical synthesis techniques, available through in-house programs, vendors, or contract research organizations (Dandapani et al., 2012). Conversely, synthesizable libraries exist as digital collections of compounds designed *in silico*, considered feasible to synthesize using known chemical reactions and commercially available reagents (Saldívar-González et al., 2020; Sadybekov and Katritch, 2023).

Several specialized categories have emerged to address specific drug discovery needs. Fragment libraries consist of low molecular weight compounds (typically <300 Da) with minimal hydrogen bond donors/acceptors, low lipophilicity, and few rotatable bonds (Dandapani et al., 2012). Lead-like libraries contain compounds with properties desirable for drug candidates, designed with a balance between structural diversity and drug-like properties (Dandapani et al., 2012; Saldívar-González et al., 2020). Natural product libraries comprise compounds derived from natural sources, providing valuable structural diversity and novel scaffolds for targeting macromolecule interactions (Dandapani et al., 2012; Heinzke et al., 2024).

Computationally generated libraries, exemplified by the GDB-17 library (160 billion molecules) and CHIPMUNK library (95 million compounds), enable cost-effective exploration of vast chemical spaces (Saldívar-González et al., 2020; Korablyov et al., 2024). While offering flexibility in design and novel structures, these face challenges including uncertainty in predicted properties and potential synthetic inaccessibility (Dandapani et al., 2012; Sadybekov and Katritch, 2023; Popova et al., 2018).

### 1.3 Filters and assessment criteria

Drug-likeness assessment primarily relies on established parameters, with RO5 setting fundamental criteria for oral bioavailability, including molecular weight under 500 Daltons, CLogP less than 5, and specific limits on hydrogen bond donors and acceptors (Dandapani et al., 2012; Ress et al., 2004). Additional guidelines have emerged for specialized applications, such as the “rule of 3” for fragment-based design and “rule of 2” for reagents, providing more targeted parameters for different molecular categories (Saldívar-González et al., 2020).

ADMET (absorption, distribution, metabolism, excretion, and toxicity) properties form a crucial component of molecular assessment (Dandapani et al., 2012; Saldívar-González et al., 2020; Chang et al., 2023). Optimal passive membrane absorption correlates with logP values between 0.5 and 3, while metabolism considerations focus particularly on cytochrome P450 interactions. Toxicity evaluation encompasses various factors, including cardiac risks through hERG channel binding, with specific attention paid to identifying pan-assay interference compounds (PAINS) to avoid false positives in biological assays.

Synthetic feasibility evaluation employs metrics such as the synthetic accessibility score (SAS), where scores above 6 indicate potentially challenging synthesis (Popova et al., 2018). Structural properties assessment includes molecular complexity measures, examining features such as chiral centers and sp2:sp3 hybridization ratios, alongside diversity analysis using molecular fingerprints and clustering algorithms (Dandapani et al., 2012).

The integration of adverse drug reaction (ADR) databases has emerged as an additional filtering criterion, enhancing toxicity predictions. Databases such as DrugCentral provide comprehensive structural and pharmacological details for early safety assessment (Halip et al., 2023). Deep learning models trained on data from Open TG-GATEs and FAERS enable ADR likelihood prediction (Mohsen et al., 2020), while the SIDER database offers drug-ADR pairs from FDA drug labels for validation (Ietswaart et al., 2020). These resources, combined with machine learning approaches, facilitate more accurate correlation of structural features with specific adverse effects, particularly through analysis of properties aligned with traditional drug-likeness criteria (Halip et al., 2023). Feature selection methods and random forest models have achieved significant improvements in ADR detection, with some studies reaching 100% accuracy for specific compounds (Liu and Aickelin, 2021).

### 1.4 Limitations of current approaches and emerging solutions

Traditional molecular filters, while valuable, often oversimplify molecular behavior in biological systems (Dandapani et al., 2012). ADMET prediction models frequently demonstrate reduced accuracy when based on computational rather than experimental data (Dandapani et al., 2012; Patel et al., 2020). These models particularly struggle with non-traditional molecules like macrocycles and PROTACs, partly due to insufficient high-quality training data. The PDBbind (Wang et al., 2005) database, for instance, inadequately represents “negative space” or suboptimal interactions, limiting its utility in predicting general binding behaviour (Sadybekov and Katritch, 2023; Vamathevan et al., 2019).

Rigid adherence to conventional filters can exclude promising compounds with unique properties that might prove effective, particularly for non-oral drugs or specific targets (Dandapani et al., 2012). This narrow focus on conventional “drug-like” space reduces the possibility of discovering novel scaffolds or chemotypes (Saldívar-González et al., 2020; Ress et al., 2004). Current approaches often overlook crucial molecular complexity factors such as three-dimensionality, chirality, and sp2:sp3 hybridization ratios (Dandapani et al., 2012).

A significant limitation emerges with newer drug modalities like macrocycles and PROTACs. Traditional molecular descriptors, developed for conventional small molecules, fail to capture features of macrocycle chemotypes relevant to their pharmacological behavior (Viarengo-Baker et al., 2021). For macrocycles, computed parameters like clogP often do not reflect true conformation-dependent lipophilicity, and traditional rules about rotatable bonds become questionable when applied to macrocyclic structures (Viarengo-Baker et al., 2021). Successful macrocycles often achieve oral bioavailability through “chameleonicity,” adapting their conformations to different environments (Garcia Jimenez et al., 2023), as exemplified by cyclosporin A (Kingwell, 2023).

PROTACs present additional challenges due to their heterobifunctional nature and large molecular weight (700–1,100 Da) (An and Fu, 2018). Their size provides more opportunities for metabolic attack (An and Fu, 2018), and their optimization requires focus on the whole molecule rather than individual components (Weng et al., 2021). Critical considerations include linker design, which affects entropy, selectivity, activity, and permeability (Weng et al., 2021). These compounds may bury up to 800–900 Å2 of ligand surface area when binding to their target, approaching protein-protein interface areas (Doak and Kihlberg, 2017).

The field is adopting various strategies to address these limitations. Chemical space exploration is expanding through combinatorial chemistry, DNA-encoded libraries, and virtual libraries of on-demand compounds (Sadybekov and Katritch, 2023; Korablyov et al., 2024; Gottipati et al., 2020). Machine Learning (ML) and Artificial Intelligence (AI) enable *de novo* design of novel scaffolds (Chang et al., 2023; Popova et al., 2018; Gottipati et al., 2020; McNaughton et al., 2022), while fragment-based approaches provide systematic methods for developing new molecules (Dandapani et al., 2012; Ress et al., 2004). Target-focused libraries leverage structural data and ligand knowledge to improve hit rates for specific protein families (Dandapani et al., 2012; Harris et al., 2011). Performance diversity strategies, selecting compounds based on assay results rather than chemical diversity alone, are showing promise (Wawer et al., 2014). Advanced computational methods, including molecular dynamics, DFT, and MMPBSA/MMGBSA/MMBAPPL, provide more sophisticated analysis capabilities (Chang et al., 2023). New approaches emphasize synthetic accessibility assessment (Popova et al., 2018; Ivanenkov et al., 2023) and improved scoring functions for virtual screening (Sadybekov and Katritch, 2023), while hybrid strategies combine computational methods with experimental validation to enhance library design effectiveness.

New assessment criteria are emerging to address these challenges. For macrocycles, modified rules suggest maintaining HBD ≤7 combined with either MW < 1,000 Da, cLogP >2.5, or TPSA <300 Å2 (Garcia Jimenez et al., 2023). PROTAC assessment requires new metrics, with fa × fg = 0.25 suggested as a minimum threshold for drug-likeness (Hornberger and Araujo, 2023). Success stories like ARV-110 and ARV-471, which entered phase I clinical trials in 2019, demonstrate the potential of these approaches despite breaking traditional rules (Békés et al., 2022; Blanco and Gardinier, 2020).

Recent advances in ADR prediction models offer potential solutions to these limitations. Random Forest models can now predict drug-ADR and target-ADR associations using *in vitro* secondary pharmacology data (Ietswaart et al., 2020), while deep learning frameworks like DeepSide utilize gene expression profiling experiments and chemical structures to predict ADRs (Uner et al., 2019). These approaches enable early identification of potential safety issues, allowing for structural modifications to reduce interactions with targets linked to severe ADRs (Ietswaart et al., 2020). The integration of multiple data types, from chemical structures to literature mining, has enhanced the predictive power of these models (Mohsen et al., 2020).

### 1.5 Role of artificial intelligence

AI and ML are revolutionizing library design and selection through multiple avenues (Patel et al., 2020). At the core of these advances lies the crucial aspect of molecular representation, where deep learning algorithms perform feature learning or representation learning, contrasting with traditional feature engineering approaches (Chuang et al., 2020). The effectiveness of these representations depends on key considerations: expressiveness to capture chemical space diversity, parsimony to maintain compactness without losing critical information, and invariance to ensure consistent representation regardless of atom numbering (Chuang et al., 2020).

When dealing with high-dimensional chemical descriptor spaces, several challenges emerge. The empty space phenomenon results in sparse dataset coverage, while the vanishing sphere volumes and distance concentration effects can complicate meaningful molecular comparisons (Reutlinger and Schneider, 2012). To address these challenges, various dimensionality reduction and feature extraction methods are employed. These include Principal Component Analysis (PCA) for uncorrelated variable transformation, Kernel PCA for nonlinear relationship analysis, and advanced techniques like symmetric encoder networks, self-organizing maps (SOM), and stochastic proximity embedding (SPE) (Reutlinger and Schneider, 2012; Sarkar et al., 2023).

Enhanced virtual screening utilizing deep learning models enables efficient analysis of large chemical spaces and improved prediction of ligand properties. Machine learning facilitates *de novo* design through generative models and reinforcement learning, creating novel molecules with desired properties and overcoming existing library limitations (Gottipati et al., 2020; McNaughton et al., 2022).

Recent developments in AI have expanded to include sophisticated ADR prediction models. Deep learning architectures trained on drug chemical structures and gene expression profiles can now predict adverse reactions with unprecedented accuracy (Uner et al., 2019). These models, integrated with databases like FAERS and SIDER, provide comprehensive safety assessments early in the drug development process (Mohsen et al., 2020; Ietswaart et al., 2020). The success of these approaches is evidenced by models achieving high accuracy in detecting major ADRs, particularly when combining multiple data sources and advanced feature selection methods (Liu and Aickelin, 2021).

Generative models, such as REINVENT (Loeffler et al., 2024), have become particularly instrumental in creating novel, synthesizable compounds by exploring vast chemical spaces beyond traditional limitations (Chang et al., 2023; Patel et al., 2020; Loeffler et al., 2024). Based on recurrent neural networks or transformers, these models can perform multi-objective optimization, simultaneously considering factors like potency, selectivity, solubility, and ADMET properties (Popova et al., 2018). They enable scaffold hopping and linker design while incorporating synthetic feasibility predictions through reinforcement learning algorithms that navigate synthetically accessible chemical space (Sadybekov and Katritch, 2023; Gottipati et al., 2020).

These advances aid in predicting ADMET and pharmacokinetic properties, guiding hit-to-lead optimization through QSAR models, and supporting target identification through omics data analysis (Sadybekov and Katritch, 2023; Patel et al., 2020). However, careful consideration must be given to the application of dimensionality reduction methods, as their misuse can lead to erroneous results and misinterpretation, particularly in hit finding and hit-to-lead optimization stages of early drug discovery (Reutlinger and Schneider, 2012). When properly implemented and combined with fragment-based drug discovery approaches and ADR prediction models, these systems provide a comprehensive framework for developing safer and more effective drugs (Korablyov et al., 2024; Ietswaart et al., 2020).

## 2 Traditional approaches to molecular library development

The efficient exploration of chemical space for drug discovery necessitates robust approaches for generating and organizing molecular libraries. Here, we present two complementary methodologies developed by our group: a chemical template-based generation system and a curated molecular database, each addressing different aspects of the drug discovery pipeline.

### 2.1 Chemical template-based generation system

We developed a comprehensive chemical template library comprising 160 distinct chemical moieties, categorized into rings, sidechains, and linkers. This modular system enables the sequential construction of both known and novel molecular structures through systematic combination and arrangement of these template elements (Latha et al., 2004). The methodology incorporates a structured workflow for molecule generation, optimization, and evaluation against target proteins.

The system’s implementation involves several key steps: initial molecule generation through template combinations, structural optimization of the generated molecules, molecular docking against target proteins, and subsequent scoring and ranking of potential candidates. This approach has been successfully implemented in the Sanjeevini software platform, facilitating active-site directed lead design (Jayaram et al., 2006; Jayaram et al., 2012).

However, during implementation, we identified a significant limitation: the disparity between computational feasibility and synthetic accessibility. Specifically, molecules that can be readily generated *in silico* may present substantial challenges for practical synthesis *in vitro*. This observation prompted the development of complementary approaches focused on curated molecular databases (Latha and Jayaram, 2005).

### 2.2 NRDBSM: a curated database for virtual screening

To address the limitations of template-based generation, we developed the Non-Redundant Database of Small Molecules (NRDBSM), specifically designed to facilitate virtual high-throughput screening (vHTS). This database represents a carefully curated collection of approximately 17,000 compounds, each selected based on stringent physicochemical criteria and optimized for lead-like characteristics (Shaikh et al., 2007; Shaikh et al., 2012).

The database construction prioritizes compliance with established drug-likeness parameters, including Lipinski’s Rule of Five and additional criteria crucial for evaluating solubility, membrane permeability, and transport characteristics. Key molecular descriptors used in the curation process include molecular weight, hydrogen bond donor and acceptor counts, partition coefficient (logP), and molar refractivity.

A distinctive feature of NRDBSM is its uniform distribution of physicochemical parameters, deliberately deviating from the typical normal distribution observed in conventional databases. The parameters are distributed across carefully selected ranges: logP values from −1.0 to 6.0, molar refractivity spanning 40 to 130, molecular weights between 150 and 480, hydrogen bond donors from 0 to 3, and hydrogen bond acceptors from 2 to 9. This distribution strategy optimizes the coverage of chemical space while maintaining drug-like characteristics.

The compounds in NRDBSM are characterized by simplified molecular structures, conservative molecular weights, minimal ring systems, controlled numbers of rotatable bonds, and moderate hydrophobicity. This intentional simplicity facilitates their prospective evolution into drug-like compounds post-vHTS, allowing for systematic structural refinement and controlled complexity augmentation (Shaikh et al., 2007; Shaikh et al., 2012).

The database incorporates a comprehensive search engine enabling users to query and filter molecules based on multiple physicochemical parameters. This functionality supports both independent virtual screening campaigns and targeted searches within larger molecular datasets, effectively streamlining the early stages of drug discovery by identifying promising candidates while minimizing subsequent optimization challenges.

These complementary approaches - template-based generation and curated database development - provide researchers with versatile tools for exploring chemical space in drug discovery. While the template-based system offers flexibility in molecular design, NRDBSM ensures practical applicability through careful curation and optimization of physicochemical properties.

### 2.3 IDRs as targets and their limitations

Intrinsically Disordered Regions (IDRs) represent an emerging class of drug targets that challenge traditional small molecule screening approaches. These regions, characterized by their structural flexibility, play crucial roles in protein-protein interactions and are frequently associated with disease states, making them attractive therapeutic targets (Han et al., 2023; Wang et al., 2023). The structural plasticity of IDRs enables them to interact with multiple partners through Short Linear Motifs (SLiMs), promoting various biological processes including cell signaling and protein modification (Han et al., 2023).

In small molecule screening, IDRs present unique opportunities and challenges. The dynamic nature of IDP-ligand interactions, where small molecules can interact with multiple sites simultaneously, necessitates modified screening approaches (Wang et al., 2023). IDP drug virtual screening (IDPDVS) has emerged as an efficient strategy, employing conformation sampling, clustering, and selection of druggable conformations to identify potential binding molecules (Ruan et al., 2021). This approach has proven particularly valuable for IDPs without known active small-molecule ligands.

Several computational methods enhance IDP-targeted drug discovery. Ensemble-based drug discovery (EBDD) employs many-to-many scoring, evaluating multiple protein conformations against numerous ligands (Wang et al., 2023). The integration of multiple experimental techniques, including NMR, SAXS, and smFRET, with computational simulations has improved IDP model accuracy (Wang et al., 2023). Recent advances in deep learning and molecular dynamics have accelerated this field, with enhanced sampling methods enabling direct generation of IDP conformations (Wang et al., 2023).

Despite these advances, the structural flexibility of IDRs complicates traditional binding site prediction and docking approaches. However, successful examples of IDP-targeting drugs advancing to clinical trials demonstrate the feasibility of this approach (Wang et al., 2023), suggesting that integrating IDR-specific considerations into screening workflows could significantly expand the druggable target space.

## 3 Specialized knowledge-based resources

The evolution of drug discovery has been significantly enhanced by specialized databases that integrate diverse data types and provide comprehensive insights into molecular interactions. These knowledge bases serve as crucial resources for improving prediction accuracy and streamlining the drug development process through the integration of traditional knowledge, experimental data, and computational approaches.

### 3.1 BIMP database

The Bioactivity of Phytochemicals of Indian Medicinal Plants (BIMP) Database (https://scfbio.iitd.ac.in/bimp/) is a comprehensive and meticulously curated resource developed to assist researchers, scientists, and professionals in exploring the therapeutic potential of India’s extensive medicinal flora. By bridging the gap between traditional knowledge and modern scientific research, the BIMP Database facilitates the discovery of bioactive compounds and therapeutic properties rooted in India’s rich botanical heritage. This database is a crucial tool in advancing the understanding of medicinal plants and their role in drug discovery and development.

The BIMP Database encompasses an extensive inventory of 6,209 unique plant species and 105,909 phytochemicals. Each entry is annotated with detailed physicochemical properties and categorized into relevant compound classifications. This exhaustive resource allows researchers to systematically explore the therapeutic applications of Indian medicinal plants, providing valuable insights for both experimental and computational studies.

One of the key features of the database is the availability of molecular data in multiple formats, including SDF, PDB, XYZ, and MOL2. These formats provide both 2D and 3D representations of molecular structures, enabling detailed visualization and analysis.

Each phytochemical entry is supplemented with an extensive profile of physicochemical properties such as solubility, polarity, and molecular weight. Additionally, the inclusion of molecular descriptors provides further structural insights, allowing researchers to better understand compound behavior and bioactivity. These detailed annotations equip users with the tools needed to evaluate the potential of compounds in therapeutic contexts.

The BIMP Database also evaluates phytochemicals against widely accepted druglikeness rules, including Lipinski’s Rule of Five, Egan’s Rule, Muegge’s Rule, Ghose’s Rule, and Veber’s Rules. Compounds that violate any of these rules are flagged, offering researchers critical insights into their suitability as viable drug candidates. This feature ensures that users can efficiently screen compounds for drug development potential.

Another significant feature of the BIMP Database is its integration of both predicted and experimentally validated pharmacological targets for phytochemicals. This dual approach provides comprehensive insights into the bioactivity of compounds and aids in identifying specific therapeutic applications. By offering predicted and experimental data, the database enables researchers to make more informed decisions in their investigations of pharmacological properties.

To further support drug discovery efforts, the database includes robust tools for virtual screening, scaffold identification, and similarity searches. These tools allow researchers to evaluate compounds efficiently based on specific criteria, streamlining the identification of potential drug candidates. Moreover, the database’s search functionality supports diverse parameters, enabling users to search for compounds by ID, name, plant species, plant family, or links to external databases such as PubChem, DrugBank, FooDB, KnapSack, ChemSpider, and CAS.

The BIMP Database serves as a valuable resource for multiple sectors, including academia, the pharmaceutical industry, and healthcare. Its applications extend to facilitating novel therapeutic discoveries, supporting evidence-based medical research, informing sustainable policymaking regarding medicinal plant usage, and promoting biodiversity conservation (Chaurasia et al., 2024). By seamlessly integrating traditional knowledge with advanced scientific methodologies, the BIMP Database fosters significant advancements in natural product research, drug development, and sustainable healthcare solutions.

### 3.2 Comparative analysis of chemical libraries

The landscape of chemical libraries encompasses various specialized databases, each offering unique features and complementary strengths. While BIMP focuses on Indian medicinal flora with 105,909 phytochemicals from 6,209 plant species, other major databases like ZINC22 provide broader coverage with over 37 billion commercially available compounds (Tingle et al., 2023). This diversity in scope and focus enables researchers to access different segments of chemical space for drug discovery.

ZINC22’s strength lies in its extensive coverage of commercially available compounds, offering advanced search capabilities and pre-calculated 3D conformers for virtual screening. The database’s CartBlanche GUI facilitates analog searching, and its tranche browser allows tailored subsetting for specific project requirements (Tingle et al., 2023; Irwin et al., 2020). In contrast, BIMP’s specialization in traditional medicine-derived compounds, complete with experimentally validated targets and comprehensive physicochemical annotations, provides a unique resource for natural product-based drug discovery.

Natural product databases like COCONUT complement these resources by aggregating information from multiple sources, improving annotations, and offering specialized focus areas (Chandrasekhar et al., 2025; Sorokina et al., 2021). While COCONUT combines data from 53 openly accessible natural product databases, specialized databases like NPAtlas focus on microbial natural products, and others like NuBBEDB and KNap-Sack concentrate on phytochemicals (Sorokina et al., 2021).

Each database offers distinct advantages in data organization and accessibility. ZINC22 provides rapid lookup of molecular properties and regular updates, with 90% of catalogs refreshed every 90 days (Tingle et al., 2023; Irwin et al., 2020). BIMP’s strength lies in its detailed physicochemical profiling, multiple molecular format availability (SDF, PDB, XYZ, MOL2), and integration of both predicted and experimental target data.

Notably, analysis of druggability across these databases reveals interesting patterns. In BIMP’s collection of over 100,000 phytochemicals, 33% conform to all major druggability rules (Lipinski, Ghose, Veber, Egan, Muegge’s), while 72% satisfy at least one rule. These proportions are relatively high compared to databases of chemically synthesized compounds, suggesting that natural product libraries might offer a richer source of drug-like molecules. This observation aligns with the historical success of natural products in drug discovery and their evolutionary optimization for biological interactions.

Together, these resources create a complementary ecosystem for drug discovery, combining commercial availability, natural product diversity, and traditional medicine knowledge. The higher druggability ratio in natural product databases like BIMP provides an additional strategic advantage for drug discovery efforts, particularly when seeking novel scaffolds with inherent biological relevance.

### 3.3 Integration of openTargets for enhanced prediction

The Open Targets Platform, an open-source knowledge base integrating data from 23 independent public sources, offers valuable insights for drug target identification and prioritization (Buniello et al., 2025). This resource uniquely combines multiple data types: genetic associations, somatic mutations, transcriptomics, pathway biology, and critically, information about approved drugs and their targets (Han et al., 2022; Koscielny et al., 2017). For approved pharmaceuticals, the platform provides extensive molecular attributes and target information, enabling more accurate prediction models through validated drug-target pairs (Koscielny et al., 2017).

The platform’s comprehensive architecture supports multiple prediction enhancement strategies. At the molecular level, it enables the creation of three-dimensional data tensors comprising gene targets, diseases, and evidence attributes (Ye et al., 2024). This integration has demonstrated significant improvements in prediction accuracy, particularly when combining target tissue specificity with functional interactions (Buniello et al., 2025). The ML-GPS (machine learning-assisted genetic priority score) framework exemplifies this approach, utilizing predicted phenotypes to enhance target identification for chronic diseases. This method has substantially expanded our understanding of drug-target relationships, supporting over 15,000 previously unvalidated gene-disease associations and identifying promising targets such as LRRK2 inhibitors for Parkinson’s disease (Chen et al., 2024).

Gene expression data within OpenTargets provides an additional layer for screening refinement. By incorporating expression profiles with molecular attributes of successful drugs, prediction models can better account for both tissue-specific and cell-type specific effects. This granular understanding of cellular responses enables more precise predictions of drug effects across different cellular contexts and tissues. The integration has proven particularly valuable for target validation and novel indication discovery, although challenges remain in normalizing heterogeneous data sources and managing computational resources for large-scale expression analysis. Despite these limitations, the combined use of validated drug-target pairs and multi-level expression data has demonstrably improved prediction accuracy, with some studies reporting significant increases in both AUROC and AUPRC metrics (Ye et al., 2024).

## 4 Advanced computational methods for screening

**FIGURE 2:**
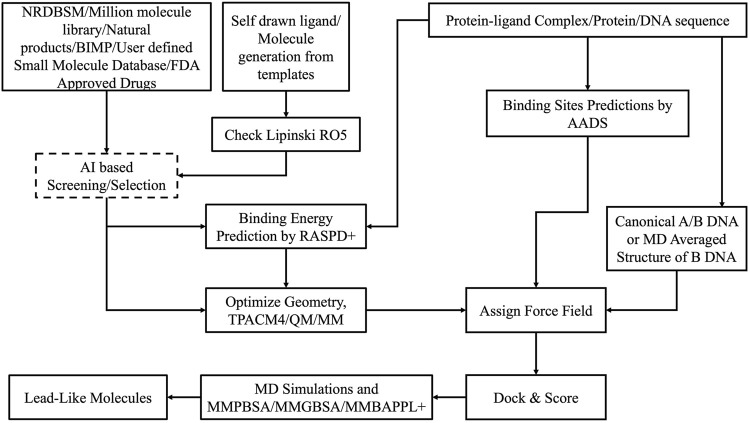
Workflow and architecture of *Sanjeevini.*

**FIGURE 3:**
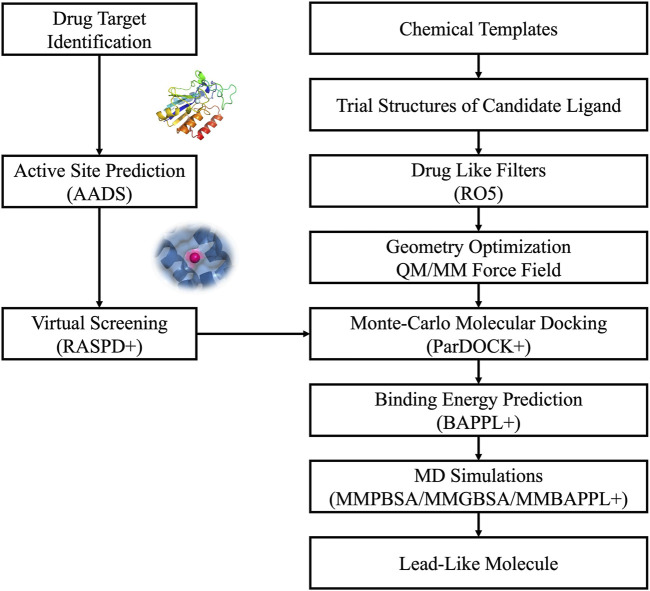
The *Sanjeevini* pathway for active site directed lead compound design *in silico*.

Virtual screening of extensive chemical libraries targeting protein binding sites is a pivotal stage in modern drug discovery. This involves computational docking of ligands into protein binding sites to estimate their binding affinities. Traditional docking methods often generate multiple poses for ligands, leading to significant computational costs and challenges in accurately predicting protein-ligand binding affinities. To address these issues, advanced computational methods like RASPD+ (Holderbach et al., 2020) and BAPPL+ (Soni et al., 2020) have been developed, building on earlier versions of our CADD/Sanjeevini Pipeline (Figures 2, 3), which established foundational approaches for bracketing drug-like compounds from templates or databases (Jayaram et al., 2006; Jayaram et al., 2012).

### 4.1 RASPD+

RASPD+ represents a pre-filtering approach designed to prioritize ligands efficiently in drug discovery workflows. By leveraging machine learning (ML) models and physicochemical descriptors that are independent of ligand conformation, RASPD+ overcomes the limitations of traditional docking methods. Unlike conventional approaches, RASPD+ does not require the generation of ligand poses, focusing instead on pose-invariant descriptors of ligands and protein binding pockets. This pose-independent methodology reduces computational costs significantly while maintaining strong predictive performance.

The ML models employed in RASPD+ are trained on the PDBbind dataset, enabling accurate prediction of protein-ligand binding affinities. When benchmarked against its predecessor and traditional scoring functions, RASPD+ demonstrates superior regression performance across multiple test datasets. These advancements make RASPD+ an ideal tool for pre-screening compound libraries in pharmaceutical research. Its ability to prioritize compounds rapidly without compromising accuracy expedites the identification of promising drug candidates, offering a highly efficient solution for early-stage drug discovery.

Performance evaluations of RASPD+ reveal its consistent and reliable regression performance, underscoring its potential to streamline the identification of prospective leads from extensive chemical libraries (Holderbach et al., 2020). This innovative method represents a significant step forward in computational drug discovery, combining computational efficiency with robust predictive capabilities.

Performance evaluations of RASPD+ reveal its consistent and reliable regression performance, underscoring its potential to streamline the identification of prospective leads from extensive chemical libraries (Holderbach et al., 2020). This innovative method represents a significant step forward in computational drug discovery, combining computational efficiency with robust predictive capabilities. The practical utility of RASPD+ is demonstrated through its successful implementation in various drug discovery projects. For instance, in the Dhanvantari platform, RASPD+ enables rapid screening of small molecule libraries against target protein active sites (Bhat et al., 2020). Its efficiency was particularly evident in a large-scale screening effort, where it successfully processed a million-molecule library against an identified site in HBsAg (Kiruthika et al., 2021), highlighting its capability to handle extensive chemical libraries while maintaining computational efficiency.

### 4.2 BAPPL+

BAPPL+ is an advanced scoring function designed to predict the binding affinities of protein-ligand (PL) complexes with enhanced accuracy. Evolved from earlier scoring functions such as BAPPL and BAPPLZ, BAPPL+ incorporates machine learning to improve prediction reliability. This new scoring function is particularly versatile, accommodating both metallo and non-metallo PL complexes, thus expanding its applicability in structure-based drug design.

The performance of BAPPL+ is underpinned by an enlarged and diverse training dataset, contributing to its enhanced predictive capabilities. It achieves a high Pearson correlation coefficient of approximately 0.76 with low standard deviations, demonstrating its reliability and precision in predicting binding affinities. These results surpass traditional scoring methods, positioning BAPPL+ as a robust tool for ranking drug candidates effectively.

BAPPL+ has been rigorously evaluated against state-of-the-art scoring systems, consistently exhibiting superior efficacy in predicting binding affinities. While its overall performance is robust, evaluations of target-specific proteins reveal certain limitations that provide opportunities for further refinement. These insights pave the way for iterative improvements, ensuring that BAPPL+ remains a dependable and precise framework for evaluating candidate compounds. The versatility of BAPPL+ is exemplified through its integration with various computational methods. It effectively calculates overall binding free energies of protein-inhibitor complexes throughout MD simulations, and can be seamlessly combined with molecular docking, quantum mechanical calculations, and molecular dynamics simulations to provide comprehensive understanding of inhibitor binding mechanisms (Kiruthika et al., 2021).

By accurately predicting binding affinities, BAPPL+ facilitates the ranking of drug candidates, streamlining the drug discovery process for both metallo and non-metallo protein targets (Soni et al., 2020; Jain and Jayaram, 2005; Jain and Jayaram, 2007). Its integration of machine learning and comprehensive dataset training, coupled with its proven applications in complex computational workflows, underscores its potential as a transformative tool in computational drug discovery, driving innovation in the identification and optimization of therapeutic compounds.

### 4.3 Molecular property predictor

**FIGURE 4:**
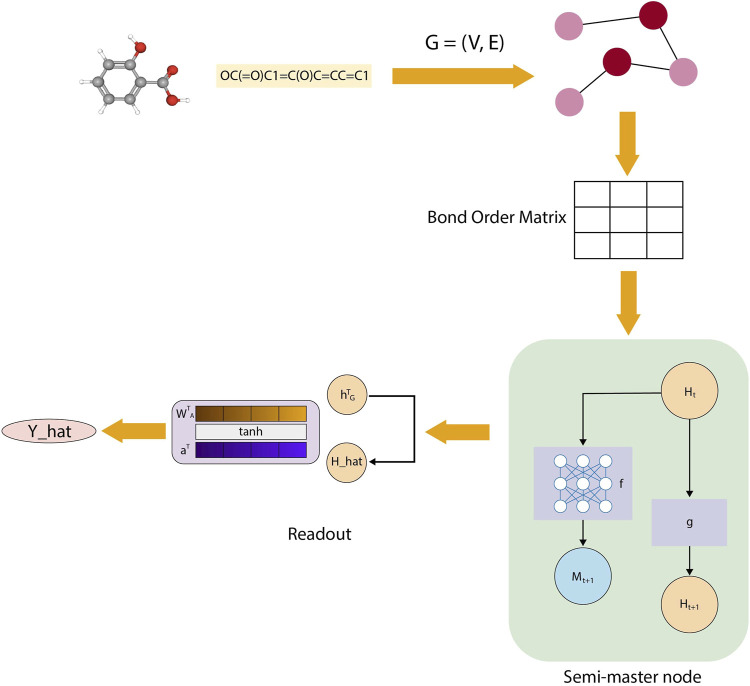
Workflow schematic of the molecular modeling pipeline. The process begins with molecular graph construction, followed by generation of a bond order matrix. A semi-master node is then incorporated to enhance information flow across the molecular structure. Finally, the Message Passing Neural Network (MPNN) processes this enhanced representation to generate predictions. This stepwise approach enables comprehensive molecular analysis while maintaining computational efficiency.

MolPropPrep (MP2) takes advantage of a novel “bond order” matrix representation of SMILES notation and utilizes the message passing neural networks (MPNNs) with a built-in semi master node to predict 15 different physico-chemical properties such as HOMO-LUMO energy gaps, dipole moments, zero-point vibrational energies (Brahmavar et al., 2024). With the introduction of semi master node in the MPNN network, one can reverse engineer the possible contributions of various functional groups to the druglikeness of small molecules. With the current implementation of this architecture and “Bond order” matrix (Figure 4), MP2 could achieve an average error ratio of 0.61, across all the predicted properties, which is an order of magnitude better than the state-of-the-art tools.

### 4.4 AI driven methods for RNA-Small molecule interactions

While traditional computational drug design has primarily focused on proteins, recent advances in AI have enabled effective screening of small molecules targeting RNA structures. Structure-based drug design (SBDD) targeting RNA presents unique challenges due to RNA’s conformational plasticity and dynamic nature, making sequence information alone insufficient for accurate predictions (Kozlovskii and Popov, 2021).

Several AI-driven approaches have emerged to address these challenges. BiteNetN, a pioneering structure-based deep learning method, effectively detects binding sites in nucleic acid structures, working with arbitrary nucleic acid complexes to demonstrate state-of-the-art performance (Kozlovskii and Popov, 2021). CplxCavity employs a two-step process, first determining surface cavities using atomic coordinates, then utilizing machine learning to predict binding sites (Pan, 2023). Additionally, geometric deep learning methods using RNA-ligand Surface Interaction Fingerprinting (RLASIF) have shown excellent performance in characterizing binding affinity through molecular surface features (Xia et al., 2025).

RNA-specific considerations have led to specialized prediction tools. RSAPred offers quantitative models for predicting RNA-small molecule binding affinity across six RNA subtypes, incorporating RNA sequence-based and small molecule structure-based features (Krishnan et al., 2024). DrugPred_RNA, though trained on protein pockets, successfully identifies druggable RNA binding sites using descriptors applicable to both RNA and protein binding sites (Rekand and Brenk, 2021).

The development of these tools account for unique characteristics of RNA-binding compounds, which typically exhibit lower octanol-water partition coefficients, greater topological polar surface areas, and more hydrogen bond donors and acceptors compared to protein-binding compounds (Childs-Disney et al., 2022). Despite these advances, the field faces limitations due to the relatively small number of available RNA structures for training deep learning models (Kozlovskii and Popov, 2021). However, continued development of AI methods, combined with experimental techniques like molecular dynamics simulations, promises to enhance our ability to predict and optimize RNA-small molecule interactions.

### 4.5 Screening approaches for multi-molecule and complex systems

The complexity of biological systems often necessitates considering multiple molecules and protein complexes in screening approaches. Recent advances in computational methods have made it feasible to predict drug combination effects and protein complex interactions efficiently.

Drug combination prediction has evolved into both classification and regression tasks, with deep learning models demonstrating superior performance in handling large High-Throughput Screening (HTS) datasets (Liu et al., 2023). Sequential Model Optimization (SMO) methods iteratively adapt to new observations, identifying highly synergistic combinations while reducing experimental burden compared to exhaustive searches (Bertin et al., 2022). The RECOVER platform exemplifies this approach, utilizing deep neural networks to predict synergy scores based on molecular fingerprints and structural features (Bertin et al., 2022).

In the target space, protein-protein interactions (PPIs) present unique challenges due to their typically large, flat interfaces (Voet and Zhang, 2012; Koes et al., 2018). AnchorQuery, a specialized web application, enables rational structure-based design of PPI inhibitors through rapid screening of synthesizable compounds. This approach particularly focuses on anchor side chains, which form energetic hot spots at binding interfaces (Koes et al., 2018).

Virtual Screening methodologies have been adapted for Small Molecule Protein-Protein Interaction Inhibitors (SMPPII) discovery, incorporating molecular docking simulations and pharmacophore modeling (Voet and Zhang, 2012). Pharmacophore models provide abstract 3D representations of essential chemical functionalities, guiding the docking of compounds to ensure desired conformations and interactions. The comparison of PPI complexes with receptor-SMPPII structures enables visual observation of interaction mimicry by small molecule ligands (Voet and Zhang, 2012).

Challenges in these complex systems include data discrepancies from inconsistent generation processes, systematic biases limiting model generalizability, and protein mutations driving resistance (Bertin et al., 2022). Advanced models like ComboKR address these challenges by predicting drug combination response surfaces using normalized data schemes (Huusari et al., 2025). The integration of cheminformatics techniques, including structure-based virtual screening and molecular dynamics, with experimental validation has proven effective in identifying dual inhibitors (Melagraki et al., 2017), demonstrating the power of combining multiple computational approaches for complex system analysis.

## 5 Integration of domain knowledge with deep learning

The progression of scientific knowledge is typically characterized by the gradual refinement of existing theories, interspersed with revolutionary breakthroughs. Recent advancements in artificial intelligence (AI) have made the prospect of AI-based scientific assistants increasingly viable, offering the potential to accelerate routine reasoning and even generate transformative ideas. For such systems to be effective, they must incorporate concepts, relations, and hypotheses familiar to human scientists. While symbolic techniques have long been employed for hypothesis generation and testing due to their ability to reuse knowledge, modern neural-based deep learning approaches provide distinct advantages. These include significantly higher predictive performance, the ability to directly process diverse observational data, and the development of interactive systems through advancements in neural language models.

**FIGURE 5:**
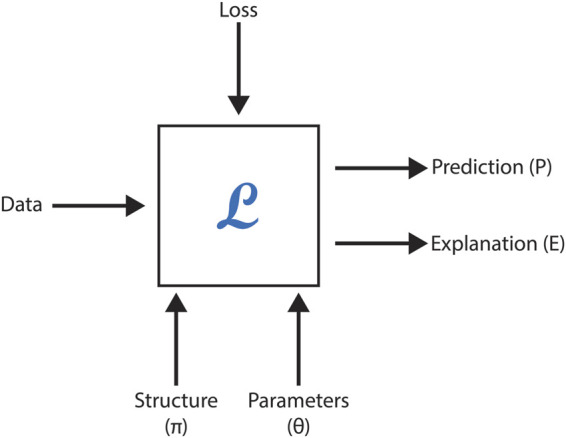
Summary of module L, which incorporates a deep network. The structure, parameters, and loss function correspond to the inputs of the deep network.

However, neural methods face challenges in leveraging formalized scientific knowledge to improve predictions, offer meaningful explanations, or ensure model correctness when generating new concepts or relationships. This section explores the feasibility of embedding formal domain knowledge into deep neural networks, demonstrating its utility through case studies focused on toxicity prediction, explanation, and molecular generation in drug discovery. By integrating symbolic knowledge with graph neural networks (GNNs), these studies highlight how hybrid approaches can enhance data representation, predictive accuracy, and overall system effectiveness. Figure 5 represents a black-box model with the following inputs and outputs, as employed in these AI-driven methodologies.

### 5.1 Case study 1: inclusion of domain knowledge to improve prediction

Understanding how domain knowledge can enhance deep learning models’ data representation is crucial in the field of machine learning. Recent research has demonstrated an innovative approach combining Graph Neural Networks (GNN) as the machine learning engine (L) with a logical inference engine ( ⊢ ) for integrating domain-specific knowledge. In a comprehensive investigation of toxicity prediction, researchers analyzed a substantial dataset comprising approximately 225,000 molecules distributed across 73 individual datasets, with each dataset containing around 3,000 molecules classified as either “toxic” or “non-toxic” based on IC50 values. The methodology incorporated domain knowledge through formal symbolic definitions of chemical concepts, including functional groups, rings, and connected structures, encompassing roughly 100 relations expressed in formalized logical notation. By employing a logical inference engine to apply these definitions, the researchers enriched molecular graphs with detailed domain-specific information. These enhanced graphs were then processed using a specialized GNN model called BotGNN to distinguish between toxic and non-toxic molecules (Dash et al., 2022).

**FIGURE 6:**
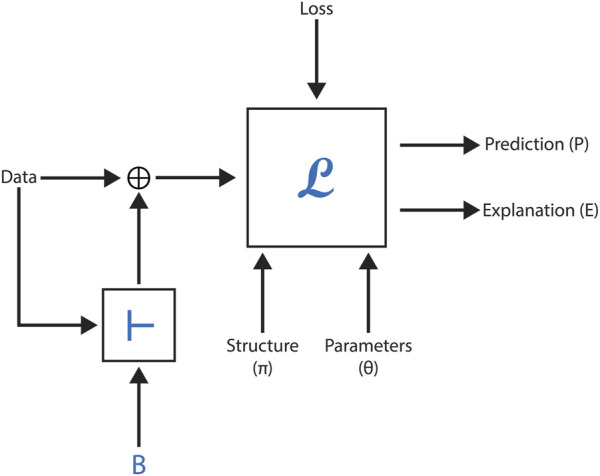
This figure shows the process of integrating domain knowledge into molecular graphs using a logical inference engine, followed by toxicity prediction with BotGNN.

Figure 6 illustrates the setup of this methodology, showcasing the flow from domain knowledge integration to GNN processing. The results demonstrate a significant improvement in predictive accuracy across most datasets. The study compares BotGNN models built using five different GNN architectures. In the comparison, baseline GNN models and state-of-the-art models using approximate background knowledge (referred to as VEGNN) are outperformed by BotGNN. This highlights the value of incorporating comprehensive background knowledge in enhancing model performance.

### 5.2 Case study 2: inclusion of domain knowledge to improve explanations

The intersection of domain knowledge and machine learning interpretability presents compelling opportunities for research advancement. A key development in this area, as demonstrated by Srinivasan et al. (2024), employs a feedforward neural network featuring a Compositional Relational Machine (CRM), which reformulates how domain knowledge structures data provided to deep learning models. This innovative framework was tested on a focused subset of data, specifically utilizing 10 datasets from a larger collection of 73 toxicity datasets, along with synthetic data containing known correct explanations as benchmarks. The research extends beyond basic chemical definitions to incorporate meta-information about chemical concepts and relations, establishing important constraints such as how rings and groups consist of sets of atoms, and how fused or connected structures require at least two structures of potentially different types.

**FIGURE 7:**
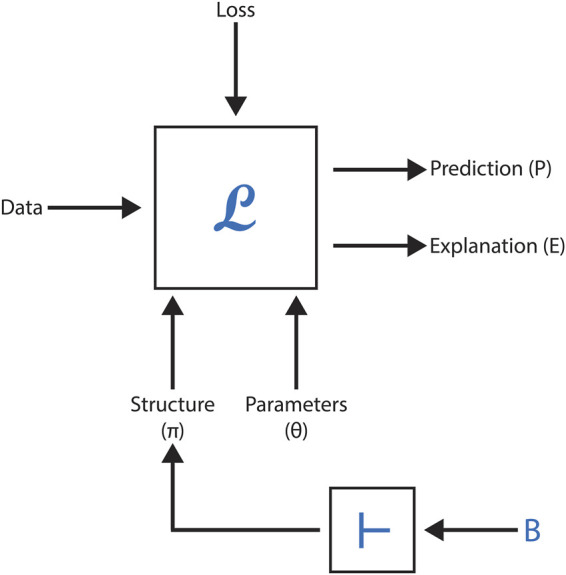
Architectural overview of the Compositional Relational Machine (CRM). The diagram illustrates how the model iteratively constructs complex features from simple features, resulting in a feedforward network structure where each non-input node represents a complex feature that can be used for toxicity prediction explanations.

**FIGURE 8:**
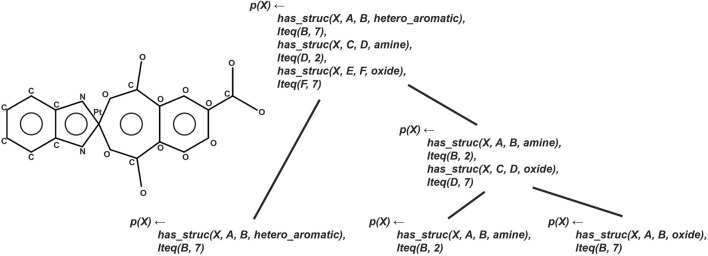
Example of a tree-like explanation structure generated by the CRM. The visualization demonstrates how activations are backtraced through the network to provide interpretable explanations of toxicity predictions, showing the hierarchical relationship between simple and complex features that contributed to the model’s decision.

Figure 7 illustrates the CRM architecture and its process of iterative feature construction. The framework utilizes provided meta-information to automatically generate a unique set of “simple features,” from which all other complex molecular features can be provably obtained through logical inference. These features are combined iteratively, with each step incorporating at least one simple feature with either another simple feature or a complex feature. The resulting feedforward network structure positions each non-input node as a complex feature, and the CRM is trained using stochastic gradient descent (SGD). The CRM serves as a proxy explainer for the BotGNN model, providing explanations by examining activations within the CRM when both models predict the same label. This approach enables a tree-like explanation structure, as the most relevant nodes can be backtraced to reveal the features involved in the CRM’s prediction. An example of this tree-like explanation structure is depicted in Figure 8.

The study effectively demonstrates how CRMs can function as interpretable proxies for more complex models like BotGNN, offering a structured approach to understanding model predictions through the lens of domain-specific features and relationships.

### 5.3 Case study 3: inclusion of domain knowledge to improve generation

**FIGURE 9:**
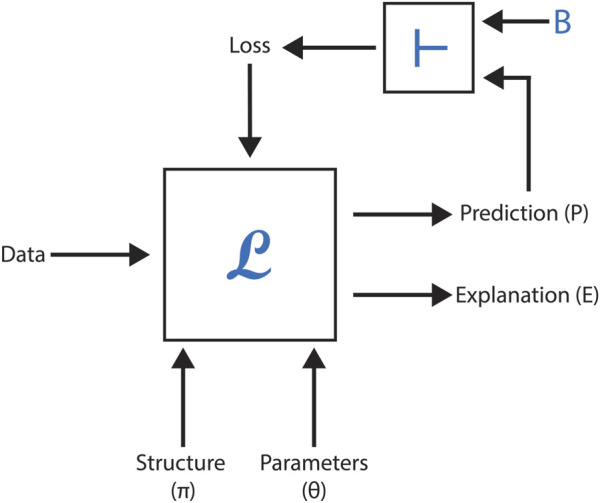
Schematic representation of the Language Models with Logical Feedback (LMLF) framework. The diagram shows the integration of domain knowledge into the model’s loss function, highlighting the feedback loop mechanism that iteratively refines the generation of molecules meeting specified logical constraints for target macromolecule binding.

Recent advances in molecular design have opened new possibilities for generating molecules with specific binding properties and physicochemical characteristics. One such approach, developed by Bhat et al. (2024), leverages domain knowledge to refine the loss function of deep learning models, specifically focusing on generating molecules capable of binding to known target macromolecules while satisfying various constraints. As illustrated in Figure 9, this method incorporates a unique feedback mechanism to enhance molecular generation.

The framework’s core component employs a Large Language Model (LLM) augmented with a novel feedback loop, known as “language models with logical feedback” (LMLF). This system iteratively identifies and reinforces constraints that guide the model toward generating “good molecules” - those meeting specified logical criteria - effectively modifying the model’s loss function through indirect means. When tested on established benchmarks for Janus kinase inhibition, the LMLF approach demonstrated superior performance, generating molecules with higher estimated binding affinity compared to both state-of-the-art methods and conventional LLMs without logical feedback. Notably, computational chemists provided favourable evaluations of the LMLF-generated molecules, particularly highlighting their novelty and potential efficacy.

## 6 Conclusion

The evolution of molecular libraries continues to play a pivotal role in the drug discovery process, bridging traditional methodologies with new advancements in computational and AI technologies. From the historical progression of combinatorial chemistry in the 1980s to modern AI-driven approaches, the field has demonstrated remarkable adaptability in addressing emerging challenges. Traditional approaches, such as template-based generation and curated libraries, have laid a strong foundation for molecular exploration. For instance, curated databases like NRDBSM and BIMP streamline the discovery process by offering pre-screened collections of drug-like molecules and phytochemicals, each designed to address specific research needs. These resources emphasize physicochemical properties, drug-likeness criteria, and accessibility for high-throughput virtual screening, enhancing their utility for early-stage discovery.

The integration of ML and AI into modern approaches marks a transformative step in molecular library development. These technologies not only expand the exploration of chemical space but also enable the generation of novel, synthesizable compounds with tailored properties. Tools such as RASPD+ and BAPPL+ exemplify how computational methods are advancing ligand screening and binding affinity predictions, reducing computational costs while maintaining robust accuracy. The incorporation of adverse drug reaction databases and OpenTargets data has further enhanced prediction efficacy, while new approaches for screening RNA targets and multi-molecule combinations demonstrate the field’s expanding scope.

Looking ahead, emerging trends signal a shift toward more diverse and complex molecular libraries, incorporating hybrid approaches that blend computational predictions with experimental validation. The rise of ultra-large virtual libraries, target-focused collections, and AI-driven generative models underscores the growing emphasis on innovation and efficiency in drug discovery. Additionally, the development of specialized assessment criteria for newer modalities like macrocycles and PROTACs reflects the field’s adaptability to emerging therapeutic approaches. By leveraging these advancements while acknowledging the limitations of traditional filtering methods, researchers can identify and optimize promising candidates more effectively, accelerating the path from molecular design to therapeutic application.

Ultimately, the convergence of traditional expertise, modern computational tools, and specialized knowledge bases promises to reshape the landscape of drug discovery, unlocking new opportunities for addressing complex biological challenges and improving human health.
